# Toll-like Receptor Homologue CD180 Ligation of B Cells Upregulates Type I IFN Signature in Diffuse Cutaneous Systemic Sclerosis

**DOI:** 10.3390/ijms25147933

**Published:** 2024-07-20

**Authors:** Szabina Erdő-Bonyár, Judit Rapp, Rovéna Subicz, Kristóf Filipánits, Tünde Minier, Gábor Kumánovics, László Czirják, Tímea Berki, Diána Simon

**Affiliations:** 1 Department of Immunology and Biotechnology, Clinical Center, Medical School, University of Pécs, H-7624 Pécs, Hungary; erdo-bonyar.szabina@pte.hu (S.E.-B.); subicz.rovena@pte.hu (R.S.); berki.timea@pte.hu (T.B.); simon.diana@pte.hu (D.S.); 2 Department of Rheumatology and Immunology, Clinical Center, Medical School, University of Pécs, H-7632 Pécs, Hungary; filipanits.kristof@pte.hu (K.F.); minier.tunde@pte.hu (T.M.); kumanovics.gabor@pte.hu (G.K.); czirjak.laszlo@pte.hu (L.C.)

**Keywords:** CD180, Toll-like receptor, B cells, interferon (IFN), signal transducer and activator of transcription 1, IFN-I receptor, anti-IFN-α autoantibodies, anti-IFN-ω autoantibodies, systemic sclerosis

## Abstract

Type I interferon (IFN-I) signaling has been shown to be upregulated in systemic sclerosis (SSc). Dysregulated B-cell functions, including antigen presentation, as well as antibody and cytokine production, all of which may be affected by IFN-I signaling, play an important role in the pathogenesis of the disease. We investigated the IFN-I signature in 71 patients with the more severe form of the disease, diffuse cutaneous SSc (dcSSc), and 33 healthy controls (HCs). Activation via Toll-like receptors (TLRs) can influence the IFN-I signaling cascade; thus, we analyzed the effects of the TLR homologue CD180 ligation on the IFN-I signature in B cells. CD180 stimulation augmented the phosphorylation of signal transducer and activator of transcription 1 (STAT1) in dcSSc B cells (*p* = 0.0123). The expression of IFN-I receptor (IFNAR1) in non-switched memory B cells producing natural autoantibodies was elevated in dcSSc (*p* = 0.0109), which was enhanced following anti-CD180 antibody treatment (*p* = 0.0125). Autoantibodies to IFN-Is (IFN-alpha and omega) correlated (dcSSc *p* = 0.0003, HC *p* = 0.0192) and were present at similar levels in B cells from dcSSc and HC, suggesting their regulatory role as natural autoantibodies. It can be concluded that factors other than IFN-alpha may contribute to the elevated IFN-I signature of dcSSc B cells, and one possible candidate is B-cell activation via CD180.

## 1. Introduction

The dysregulation of type I interferons (IFN-I) has been observed in several pathological conditions, including inflammatory and autoimmune diseases, chronic infections, and cancer [1,2,3,4]. The contribution of IFN-alpha (IFN-α) in the pathogenesis of autoimmune diseases was supported by the fact that IFN-α therapy of malignant tumors and hepatitis induced an increase in the prevalence of pathological autoantibodies and the development of various autoimmune diseases such as systemic lupus erythematosus (SLE), systemic sclerosis (SSc), and rheumatoid arthritis (RA) [5,6]. Moreover, in a randomized, placebo-controlled trial, a worsening of skin and lung involvement was described in early SSc patients treated with IFN-α [7]. Overexpression of IFN-regulated genes (IRGs) in peripheral blood mononuclear cells (PBMCs) and affected tissues has been demonstrated in SLE patients, which was associated with serological and clinical manifestations, disease activity, and severity [8,9]. Anifrolumab, an anti-IFN-I receptor (IFNAR) monoclonal antibody, has been already approved as treatment for SLE [9,10]. Increased expression of IRGs was also reported in SSc in PBMCs and in skin biopsies [11,12,13,14]. In addition, Brkic et al. [15] showed that elevated IFN-I signature is present from the earliest stages of SSc, even before the onset of fibrosis. Furthermore, expression levels of several IRGs correlated with the degree of skin involvement [16,17], and plasma concentrations of IFN-inducible chemokines were associated with skin, lung, and muscle involvement in SSc patients [18]. The IFN-Is include IFN-α, IFN-beta (IFN-β), IFN-epsilon (IFN-ε), IFN-kappa (IFN-κ), and IFN-omega (IFN-ω) in humans, which are pleiotropic cytokines, and, besides their antiviral, antiproliferative, and antitumor effects, they are known for their essential function in modulating both innate and adaptive immune responses [19,20]. They exert a specific influence on B-cell functions including survival, proliferation, differentiation, activation, receptor expression, antigen presentation, and cytokine and antibody production [21,22]. All IFN-I subtypes act through the same IFNAR, which is composed of the IFNAR1 and IFNAR2 subunits, and initiate a classical signaling cascade via the Janus kinase (JAK)/signal transducer and activator of transcription (STAT) pathway. The ligation of IFNAR activates the receptor-associated protein tyrosine kinase JAK1 and tyrosine kinase 2 (TYK2), which phosphorylate two cytoplasmic transcription factors, the STAT1 and STAT2, resulting in the upregulation of IRGs [1,23,24]. The presence of autoantibodies targeting several cytokines, including IFNs, has been observed in healthy individuals and autoimmune patients, which may influence the availability and activity of cytokines [25,26]. Signaling through Toll-like receptors (TLRs) acts as an important regulator of IFN-I response. Activation through TLRs not only induces IFN-I production but also influences the signaling cascade followed by IFN-I binding affecting the phosphoinositide 3-kinase (PI3K)/Akt, mitogen-activated protein kinase (MAPK), and nuclear factor kappa-light-chain-enhancer of activated B cells (NF-κB) signaling pathways [1,23,27,28]. We have previously shown that the ligation of the TLR homolog CD180 induces the phosphorylation of PI3K/Akt and NF-κB in the B cells of patients with the more severe form of SSc, diffuse cutaneous SSc (dcSSc), to a significantly lesser extent than in healthy controls (HCs) [29,30]. Stimulation via the CD180 molecule can activate B cells and stimulate their cytokine production [31]. Previously, we found that CD180 ligation had different effects on the activation and cytokine production of B cells in dcSSc and HCs, activating memory B cells to a greater extent in dcSSc B cells, whereas it could only stimulate IL-10 production by B cells in HCs but not in dcSSc B cells [30]. Based on our previous results, we hypothesized that IFN-I signaling may be modulated by CD180 signaling, and therefore the aim of this study was to investigate the effect of CD180 ligation on IFN-I signaling in dcSSc B cells.

## 2. Results

### 2.1. Patients’ Characteristics

Seventy-one patients with dcSSc were included in our studies; their detailed characteristics are shown in Table 1. The mean (SD) disease duration was 8.2 (±6.9) years based on the date of the first non-Raynaud’s symptom; the mean (SD) age at enrollment was 52.73 (±14.8) years; the mean (SD) modified Rodnan skin score (mRSS) was 15.12 (±9.8) points; and the frequent internal organ involvements were interstitial lung disease (71.8%), cardiac involvement (47.9%), and gastrointestinal involvement (32.4%).

### 2.2. Upregulated IRG Expression in dcSSc B Cells

**Figure 1:**
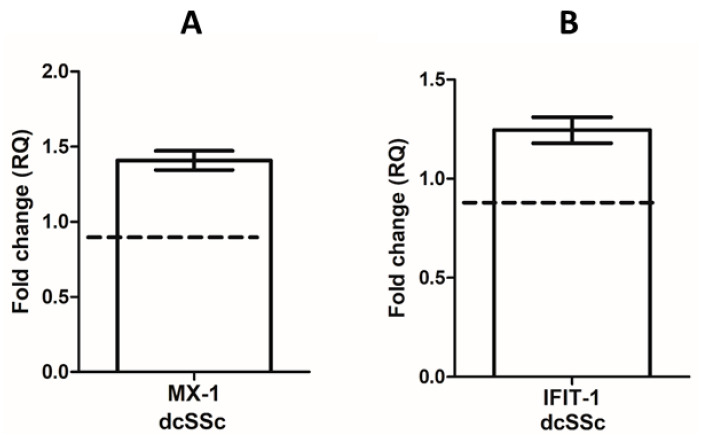
Analysis of IRGs MX-1 (**A**) and IFIT-1 (**B**) mRNA expression in purified B cells of diffuse cutaneous systemic sclerosis (dcSSc) patients (n = 3) and healthy controls (HC) (n = 3). Gene expression was normalized to HCs, and the horizontal line (value 1) represents the expression of control samples. Changes in gene expression are shown as relative quantification (RQ) values. Data are shown as mean ± standard error of the mean (SEM).

The overexpression of IRGs including Myxovirus resistance protein 1 (MX-1) and interferon-induced protein with tetratricopeptide repeats 1 (IFIT-1) has already been described in PBMCs [32,33], monocytes, and CD4 lymphocytes from SSc patients [34] but has not yet been investigated in B cells. Therefore, we analyzed the expression of two IRGs, MX-1 and IFIT-1, in purified B cells from patients with dcSSc and HCs. The upregulation of both IRGs, MX-1 and IFIT-1, was observed in dcSSc B cells compared to HCs (Figure 1).

### 2.3. CD180 Ligation Enhances the Phosphorylation of STAT1 in dcSSc B Cells

**Figure 2:**
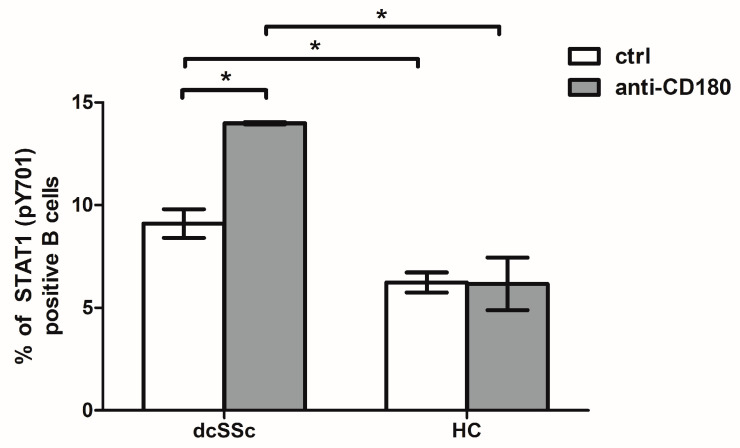
Effect of CD180 ligation on the phosphorylation of STAT1 by CD180 ligation. Changes in the phosphorylation of STAT1 (pY701) molecule in B cells of dcSSc patients (n = 3) and HCs (n = 3) after stimulation with anti-CD180 antibody (anti-CD180) or left unstimulated (ctrl) for 30 min detected as percentage of STAT1 (pY701) positive B cells by flow cytometry. Data are presented as means ± SEM. * *p* < 0.05.

The binding of IFN-I to IFNAR induces the phosphorylation of STAT1 via JAKs [1,23,24] and the overexpression of the STAT1 gene in PBMCs from SSc patients [32]. However, the elevated expression of STAT1 protein has only been reported in B cells from SLE patients [35,36]. As the increased phosphorylation of STAT1 can be the element of enhanced IFN-I signaling, we measured the phosphorylation of STAT1 in the B cells of the dcSSc patients and HCs and found that the percentage of phosphorylated STAT1 (pY701)-positive B cells was significantly higher in dcSSc B cells than in HCs (Figure 2). Since signals through TLRs can affect the phosphorylation of STAT1 [37], we investigated the effect of stimulation via TLR homologue CD180 on the activation of STAT1 in the B cells and showed that CD180 ligation significantly increased the proportion of phosphorylated STAT1 (pY701)-positive B cells only in the dcSSc patients, resulting in a significantly elevated ratio of phosphorylated STAT1 (pY701)-positive B cells in the dcSSc patients compared to the HCs under stimulated conditions (Figure 2).

### 2.4. Anti-CD180 Antibody Treatment Promotes the IFNAR1 Expression of B Cells Both in dcSSc and HCs

**Figure 3:**
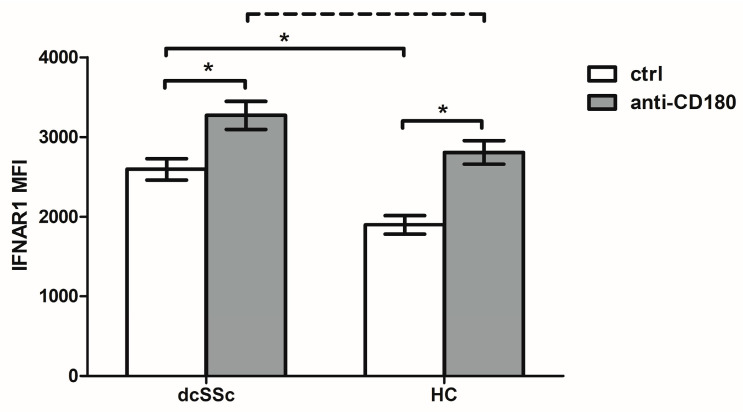
Changes in the expression of IFNAR1 in anti-CD180 antibody stimulated (anti-CD180) and unstimulated (ctrl) of total B cells from dcSSc patients (n = 4) and HCs (n = 4) measured as mean fluorescence intensity (MFI) by flow cytometry. The solid lines show significant differences (* *p* < 0.05), while the dashed line indicate tendencies (*p* < 0.1). Data are presented as means ± SEM.

The expression level of the IFNAR can also affect the IFN-I signature of cells [23]; therefore, we determined the expression of IFNAR1 in dcSSc and HC B cells. Significantly higher basal IFNAR1 expression measured as mean fluorescence intensity (MFI) was observed in the B cells of dcSSc patients compared to HCs (Figure 3). Next, to evaluate the influence of CD180 on the expression of IFNAR1, we treated the B cells with anti-CD180 antibody. The treatment significantly enhanced the expression of IFNAR1 in both dcSSc and HC B cells. In the HCs, the expression reached the levels observed in dcSSc under unstimulated conditions while it remained tendentiously higher in dcSSc than HCs (Figure 3).

### 2.5. CD180 Stimulation Increases the IFNAR1 Expression of NS B Cells to a Greater Extent in dcSSc Patients

**Figure 4:**
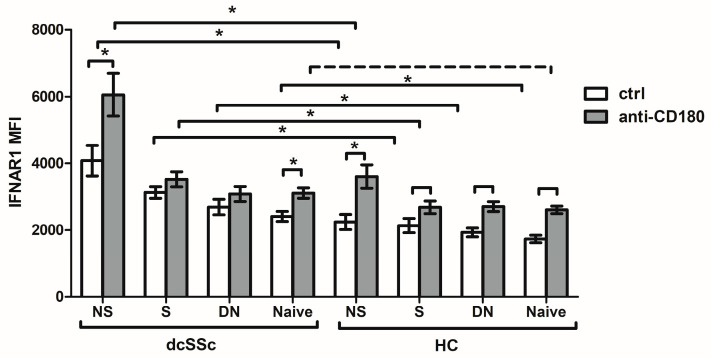
Impact of CD180 ligation on IFNAR1 expression of B-cell subpopulations in dcSSc patients (n = 4) and HCs (n = 4). The mean fluorescence intensity (MFI) of IFNAR1 of B cells was measured by flow cytometry in the four B-cell subsets defined by CD27 and IgD labeling; CD27+IgD+ non-switched memory (NS), CD27+IgD− switched memory (S), CD27−IgD− double negative (DN), and CD27−IgD+ naive B-cell subsets after anti-CD180 antibody stimulation (anti-CD180) or left unstimulated (ctrl). The solid lines show significant differences (* *p* < 0.05), while the dashed line indicate tendencies (*p* < 0.1). Data are presented as means ± SEM.

Since we found differences in IFNAR1 expression in the total B cells between the dcSSc patients and HCs, we examined the expression of IFNAR1 in the following B-cell subsets defined by CD27 and IgD labeling: CD27+IgD+ non-switched memory (NS), CD27+IgD− switched memory (S), CD27−IgD− double negative (DN), and CD27−IgD+ naive B cells. We compared the expression of the IFNAR1 of the B-cell subgroups between the dcSSc patients and HCs and found that the basal IFNAR1 expression was significantly higher in all tested B-cell subsets of dcSSc than in HCs (Figure 4). Next, we investigated the effect of stimulation with anti-CD180 antibody on the expression of IFNAR1. Ligation through CD180 significantly increased IFNAR1 expression in NS and naive B-cell subsets in dcSSc and in all examined B-cell subgroups in the HCs (Figure 4). Focusing on the differences in the expression of the IFNAR1 of the B-cell subsets after the anti-CD180 antibody treatment between the dcSSc patients and HCs, we found that the IFNAR1 expression was significantly elevated in NS and S memory B cells and tended to be elevated in the naive B cells of dcSSc patients compared to HC B-cell subsets. However, CD180 ligation enhanced the IFNAR1 expression of NS B cells to the greatest extent among the investigated B-cell subsets in both the dcSSc patients and HCs (Figure 4).

### 2.6. Autoantibodies against IFN-α and IFN-ω Are Correlated and Present at Similar Levels in dcSSc and HCs

**Figure 5:**
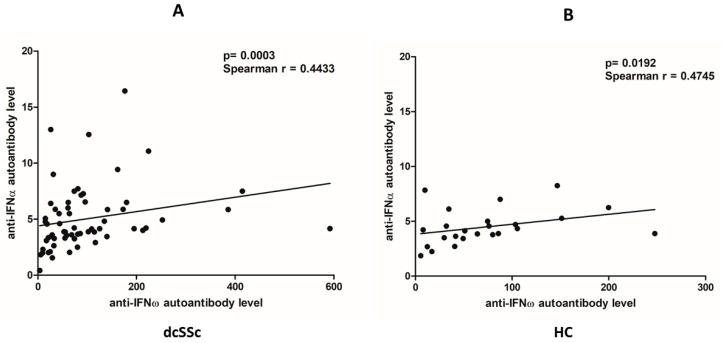
Correlation between serum levels of autoantibodies against IFN-Is. Correlation between anti-IFN-α and anti-IFN-ω autoantibody serum levels in dcSSc (n = 62) (**A**) and HC (n = 24) (**B**) measured by Luminex MAGPIX.

Anti-IFN autoantibodies are present in healthy individuals and have been described in autoimmune disease including SLE [25,26,38,39]. Since anti-IFN autoantibodies may also affect the utilization of IFN-Is [25,26], we investigated the MFI levels of autoantibodies against three IFN-Is—IFN-α, IFN-β, and IFN-ω—in the serum samples of dcSSc patients and HCs. Anti-IFN-β autoantibodies were only detectable in a small number of individuals, so statistical analysis could not be performed. No significant difference was found between the dcSSc and HC in anti-IFN-α (*p* = 0.6449, dcSSc median = 4.14, interquartile range (IQR) = 3.31–5.88; HC median = 4.33, IQR = 3.44–5.73) and anti-IFN-ω autoantibody levels (*p* = 0.782, dcSSc median = 71.06, IQR = 31.58–116; HC median = 64.72, IQR = 32.39–103.3). No significant correlation was found in dcSSc between anti-IFN-I autoantibody levels and pulmonary function test values or mRSS. Next, we examined the correlation between anti-IFN-α and anti-IFN-ω autoantibody levels in the dcSSc patients and HCs and found that they significantly positively correlated with each other in both dcSSc (Figure 5A) and HC (Figure 5B).

## 3. Discussion

Impaired immune regulation is a hallmark of SSc [40,41,42]. Overactivation of type I IFN signaling has been observed in SSc characterized by the overexpression of the IRGs and elevated serum levels of IFN-induced chemokines [12,13,43] as in SLE [4,8,9]. IFN-I signaling can be modulated by several epigenetic mechanisms, such as miRNAs [44], long non-coding RNA (lncRNA) expression [45], DNA methylation [46,47,48,49], and histone modification [50]. The hypomethylation of IRGs as a common characteristic in RA, SLE, and SSc was identified [46,47,48]. Furthermore, the hypomethylation of IRGs was detected in B cells in SLE [49]. The upregulation of IRGs has already been described in skin biopsies, PBMCs, monocytes, and CD4-positive lymphocytes from SSc patients [17,32,33,34]. However, their expression in B cells from SSc patients remains unexplored, while B cells are key players in the pathogenesis of SSc, mainly through the production of pathogenic autoantibodies and pro-inflammatory cytokines [51,52,53]. Moreover, IFN-Is are known to significantly influence these B-cell functions [21,22]. We showed the upregulation of two IRGs, MX-1 and IFIT-1, in the B cells of the dcSSc patients compared to the HCs suggesting the elevated activation of IFN-I signaling in the B cells of the dcSSc patients, which is consistent with the finding in SLE B cells [54,55]. Since the phosphorylation of STAT1 plays a key role in IFN-I signaling [23,56] and the increased gene expression of STAT1 has already been described in the PBMCs of SSc and SLE patients [32,57], we examined the phosphorylation of STAT1 in the B cells of dcSSc patients and HCs. The proportion of phosphorylated STAT1-positive B cells was significantly higher in the dcSSc patients than in the HCs, also indicating an activated IFN-I signaling in the dcSSc B cells. The established crosstalk between TLR and IFN-I signaling not only involves the canonical JAK-STAT pathway as IFNAR activation can trigger multiple signaling cascades, including PI3K/Akt, mammalian target of rapamycin (mTOR), NF-κB, and MAPKs. These downstream pathways are also used by TLRs, highlighting a strong connection between IFN-I and TLR pathway signaling [1,23,28]. We have previously shown that stimulation via CD180 affects the phosphorylation of PI3K/Akt and NF-kB in B cells to different extents in dcSSc and HC [29,30], and it has also been described previously that CD180 can utilize the p38 MAPK pathway [58]. The phosphorylation of STAT1 has been observed upon stimulation with multiple TLRs [37,59,60]. In line with this, we also found that stimulation with anti-CD180 antibody increased the percentage of phosphorylated STAT1-positive B cells only in dcSSc, resulting in an even greater difference in STAT1 phosphorylation between dcSSc and HC. This suggests that activation of B cells via CD180 may contribute to increased IFN-I signaling in dcSSc.

Pogue et al. [61] demonstrated that all blood leukocytes in healthy individuals express IFNAR1, with the highest levels in monocytes and B cells. An increased mRNA expression of IFNAR1 in whole blood from SLE patients [62] and elevated IFNAR1 protein expression in SLE B cells have been observed [63]. Similarly, we found significantly higher IFNAR1 expression in the B cells of dcSSc patients than in HCs. Treatment with anti-CD180 antibody in HC B cells increased the expression of IFNAR1 to the levels observed in dcSSc B cells in the basal state, while in dcSSc it increased to even higher levels. Interestingly, among the investigated B-cell subsets, the NS B-cell subgroup showed the highest expression of IFNAR1 in both the unstimulated and CD180-stimulated conditions in dcSSc and in HCs. Moreover, treatment with the anti-CD180 antibody enhanced the expression of IFNAR1 to the greatest extent in NS B cells. NS B cells are similar to B1 B cells with innate-like properties [64], suggesting that they are able to produce natural autoantibodies. Natural autoantibodies are polyreactive autoantibodies directed against evolutionary conserved cellular structures. They are detectable in healthy individuals having a protective role in the maintenance of self-tolerance and are important in the regulation of inflammation and autoimmune processes [65,66] but can become dysregulated in autoimmune diseases. We have already shown that stimulation via CD180 induced natural autoantibody production [67]. Autoantibodies against IFN-Is can also be considered as natural autoantibodies since they are present in healthy individuals. Consequently, anti-IFN autoantibodies were selected for investigation from the potential natural autoantibodies. Interestingly, the levels of anti-IFN-α and anti-IFN-ω autoantibodies showed a positive correlation, which may be explained by cross-reactive antibodies due to the structural similarities between IFN-α and IFN-ω [19,20]. Anti-cytokine autoantibodies have been theorized to increase in order to compensate excessive levels of target cytokines, since the binding of autoantibodies to their target cytokines can influence their availability and activity [25,68]. In agreement with this, only the neutralizing effect of high levels of anti-IFN-α autoantibodies has recently been described in SLE [38]. We found similar levels of autoantibodies against IFN-α and IFN-ω in dcSSc and HCs. A comparable level of anti-IFN-α autoantibodies to those found in HCs was not neutralizing in SLE but rather was thought to slow the elimination of cytokines from the circulation by keeping them in immune complexes, which could indicate the regulatory function of these autoantibodies [38]. The similar levels of autoantibodies against IFN-α and IFN-ω that we found in the dcSSc patients and HCs may indicate their regulatory role as natural autoantibodies.

Several lines of evidence indicate that factors other than IFN-α may contribute to the increased IFN-I signature. We have previously reported that the decreased CD180 expression in B cells in dcSSc may be the result of their activation via CD180 [29]; therefore, we can conclude that this activation could result in the upregulation of IFN-I signaling in dcSSc B cells at the level of IRG expression, STAT1 phosphorylation, and IFNAR1 expression. Furthermore, NS memory B cell activation via CD180 may contribute to the production of regulatory natural autoantibodies against IFN-I. In addition, additional stimulation through CD180 may enhance the already increased IFN-I signaling in dcSSc B cells.

## 4. Materials and Methods

### 4.1. Patients Cohort

Seventy-one patients with dcSSc were included in the study. They all fulfilled the 2013 ACR/EULAR SSc classification criteria [69] and were considered to be suffering from the diffuse form of the disease according to the classification system proposed by LeRoy et al. [70]. A complete medical history was taken for each patient, and physical examinations and laboratory tests were performed. Patients with overlap syndrome, tumors, and current infections were excluded from the study. The duration of the disease was determined from the appearance of the first non-Raynaud’s symptom. Skin thickness was assessed by mRSS [71]. Pulmonary fibrosis was characterized by the detection of fibrosis with high-resolution CT and/or decreased forced vital capacity (FVC < 80%). Cardiac involvement was defined by diastolic dysfunction or decreased left ventricular ejection fraction. Gastroesophageal involvement was established with barium swallow or esophago-gastroscopy. Controls (n = 33) were age and sex-matched HCs. All participants gave written informed consent to the study, after approval by the Hungarian National Ethics Committee (ETT TUKEB 47861-6/2018/EKU).

### 4.2. Peripheral Blood Mononuclear Cell Isolation and B-Cell Separation

PBMCs were isolated by Ficoll-Paque Plus (GE Healthcare, Chicago, IL, USA) density gradient centrifugation of peripheral blood samples from dcSSc patients (n = 9) and HCs (n = 9). The negative selection of B cells was carried out using the MACS B cell isolation kit II (Miltenyi Biotech, Bergisch Gladbach, Germany), according to the manufacturer’s instructions. The purity of the B cells was over 95%.

### 4.3. RNA Isolation, cDNA Synthesis, and qPCR for the Evaluation of MX-1, IFIT-1 Expression

To determine the MX-1 and IFIT-1 mRNA expression of total B cells in the dcSSc patients (n = 3) and HCs (n = 3), a NucleoSpin RNA XS kit (Macherey-Nagel Inc., Bethlehem, PA, USA) was used to extract the total RNA from the isolated B cells. Following the cDNA generation (High-Capacity cDNA Reverse Transcription Kit, Thermo Fisher Scientific, Waltham, MA, USA), the mRNA expression of MX-1 and IFIT-1 was determined in the total B cells of dcSSc and HCs using the SensiFAST SYBR Lo-ROX Kit (Bioline, London, UK). An Applied Biosystems 7500 RT-PCR System (Thermo Fisher Scientific, Waltham, MA, USA) was used to perform the amplifications. Gene expression was analyzed with 7500 Software v2.0.6 (Thermo Fisher Scientific, Waltham, MA, USA) and normalized to *GAPDH* (a “housekeeping” gene) as a reference. Fold changes (RQ) were calculated according to the 2^−ΔΔCT^ method.

### 4.4. Evaluation of the Phosphorylation of STAT1

For Phosflow assay in dcSSc patients (n = 3) and HCs (n = 3), 5 × 10^5^ PBMCs per condition were added onto a 96-well plate in RPMI culture medium without FBS for 1 h. Cells were then stimulated with Ultra-LEAF purified anti-human CD180 (RP105) antibody (Clone: MHR73-11) (Bio-Legend, San Diego, CA, USA) at 10 µg/mL (anti-CD180) or left unstimulated for 30 min at 37 °C. For the analysis of the STAT1 phosphorylation in the B cells of dcSSc and HC, we used anti-human CD19-FITC (HIB19, BD Biosciences, Franklin Lakes, NJ, USA) and anti-human STAT1 (pY701)–Alexa Fluor647 (4a, BD Biosciences, Franklin Lakes, NJ, USA) antibodies. Phosflow assay was performed in PBMCs according to the BD Phosflow Protocol, using BD Cytofix Fixation Buffer and BD Perm III Buffer (BD Biosciences, Franklin Lakes, NJ, USA). Briefly, after stimulation, the cells were immediately fixed with pre-warmed Cytofix Fixation buffer for 10 min at 37 °C. Following washing, the cells were permeabilized using pre-cooled Perm Buffer III for 30 min on ice. The cells were then washed three times, stained, and incubated for 30 min at room temperature. Afterwards, the cells were washed and immediately measured without fixation using a DxFlex flow cytometer (Beckman Coulter, Brea, CA, USA) and analyzed by CytExpertsoftware v2.2 (Beckman Coulter, Brea, CA, USA).

### 4.5. Flow Cytometric Analysis of IFNAR1 Expression

To examine the expression of the IFNAR1 of the total B cells and B-cell subsets in the dcSSc patients (n = 4) and HCs (n = 4), 5 × 10^5^ PBMCs were stimulated with anti-CD180 antibody or left unstimulated for 24 h at 37 °C. Next, PBMCs were labeled using a combination of the following monoclonal antibodies: anti-human CD19-Alexa Fluor 700 (SJ25C1, BioLegend, San Diego, CA, USA), anti-human IgD-PerCP (IA6-2, BioLegend, San Diego, CA, USA), anti-human CD27-FITC (L128, BD Biosciences, Franklin Lakes, NJ, USA), and anti-human IFNAR1-PE (85228, Invitrogen, Waltham, MA, USA), following the manufacturer’s protocols. Briefly, PBMCs were incubated with the appropriate antibodies for 30 min on ice, washed twice in phosphate-buffered saline (PBS) and fixed with FACSFix (0.5% PFA in PBS). The fluorescence of the labeled cells was measured using a DxFlex flow cytometer (Beckman Coulter, Brea, CA, USA) and analyzed by CytExpert software v2.2 (Beckman Coulter, Brea, CA, USA).

### 4.6. Measurement of Anti-IFN-I Autoantibodies

The MFI levels of autoantibodies against IFN-α, IFN-β, and IFN-ω in the serum samples of the dcSSc (n = 62) patients and HCs (n = 24) were determined using a MILLIPLEX Map Human Cytokine Autoantibody Expanded IgG Kit (HCYTABG-17K, Merck KGaA, Darmstadt, Germany) according to the manufacturer’s recommendations. A Luminex MAGPIX instrument (Luminex Corporation, Austin, TX, USA) was used to perform the assay, and Belysa software v1.1 (Merck KGaA, Darmstadt, Germany) was used to analyze the data.

### 4.7. Statistical Analysis

An SPSS v. 27.0 statistics package (IBM, Armonk, NY, USA) was used for statistical assessment using Student *t*-tests and Mann–Whitney U-test and Spearman’s correlation, where *p* values < 0.05 were considered significant and *p* values < 0.1 regarded as a tendency.

## Figures and Tables

**Table 1:** Patients’ characteristics.

Characteristics	dcSSc Patients (n = 71)
Age (years), mean (SD)	52.73 (14.8)
Gender (female), n (%)	58/71 (81.7%)
Disease duration ^1^ (years), mean (SD)	8.2 (6.9)
**Organ involvement**	
MRSS mean (SD)	15.12 (9.8)
Lung fibrosis ^2^, n (%)	52/71 (71.8%)
Cardiac involvement ^3^, n (%)	34/71 (47.9%)
Gastrointestinal involvement ^4^, n (%)	23/71 (32.4%)
**Antibodies**	
Anti-Scl-70+, n (%)	32/71 (45.1%)
Anti-RNA-polymerase III+, n (%)	10/71 (14.1%)

^1^ Onset of the disease was defined as the date of the first non-Raynaud’s symptom; ^2^ pulmonary fibrosis was characterized by detection of fibrosis with high-resolution CT and/or decreased forced vital capacity (FVC < 80%); ^3^ cardiac involvement was defined by diastolic dysfunction or decreased left ventricular ejection fraction; ^4^ gastroesophageal involvement was established with barium swallow or esophago-gastroscopy.

## Data Availability

Data are contained within the article.
